# Incorporating chemical sub-structures and protein evolutionary information for inferring drug-target interactions

**DOI:** 10.1038/s41598-020-62891-2

**Published:** 2020-04-20

**Authors:** Lei Wang, Zhu-Hong You, Li-Ping Li, Xin Yan, Wei Zhang

**Affiliations:** 1 College of Information Science and Engineering, Zaozhuang University, Zaozhuang, Shandong 277100 China; 2 Xinjiang Technical Institutes of Physics and Chemistry, Chinese Academy of Sciences, Urumqi, 830011 China; 3 School of Foreign Languages, Zaozhuang University, Zaozhuang, Shandong 277100 China

**Keywords:** Computational models, Data mining

## Abstract

Accumulating evidence has shown that drug-target interactions (DTIs) play a crucial role in the process of genomic drug discovery. Although biological experimental technology has made great progress, the identification of DTIs is still very time-consuming and expensive nowadays. Hence it is urgent to develop *in silico* model as a supplement to the biological experiments to predict the potential DTIs. In this work, a new model is designed to predict DTIs by incorporating chemical sub-structures and protein evolutionary information. Specifically, we first use Position-Specific Scoring Matrix (PSSM) to convert the protein sequence into the numerical descriptor containing biological evolutionary information, then use Discrete Cosine Transform (DCT) algorithm to extract the hidden features and integrate them with the chemical sub-structures descriptor, and finally utilize Rotation Forest (RF) classifier to accurately predict whether there is interaction between the drug and the target protein. In the 5-fold cross-validation (CV) experiment, the average accuracy of the proposed model on the benchmark datasets of *Enzymes*, *Ion Channels*, *GPCRs* and *Nuclear Receptors* reached 0.9140, 0.8919, 0.8724 and 0.8111, respectively. In order to fully evaluate the performance of the proposed model, we compare it with different feature extraction model, classifier model, and other state-of-the-art models. Furthermore, we also implemented case studies. As a result, 8 of the top 10 drug-target pairs with the highest prediction score were confirmed by related databases. These excellent results indicate that the proposed model has outstanding ability in predicting DTIs and can provide reliable candidates for biological experiments.

## Introduction

Drugs can regulate the physiological function of the human body, to provide guarantee for disease prevention, treatment and other aspects. More importantly, the discovery and identification of drug targets is the source of drug research, which plays a key role in the success of drug development. The complexity of the etiologies of most diseases leading to disease-related genes or proteins may be potential drug targets, but because of target specificity, robustness of biological networks and other factors, the number of newly developed drugs does not rapidly increase with the development of proteomics and chemical genomics. So far, only a small number of targets in the human genome, in which the total number of pharmacological interest is about 6000 to 8000, have been confirmed to be associated with approved drugs^1–4^. As the experiment-based method having the disadvantage of high cost, time consuming and limitations of small-scale in identifying drug-target interactions, researchers try to mine drug-related targets in the whole genome using computational-based methods^5–11^.

At present, researchers have designed many computational-based models to analyze and predict drug-target interactions (DTIs)^12–18^. For example, Yamanishi *et al*. designed a model based on statistical algorithm to predict potential DTIs, which makes full use of chemical structure and genomic sequence information. In case of unnecessary to know the 3D structure information of the protein, the DTIs is formalized as the bipartite graph^19^. He *et al*. proposed a novel clustering model CNMMA, which uses the edge structure and multi-modality attributes associated with vertices to discover network clusters, and obtains an optimal latent matrix to represent the cluster membership for each vertex in the network^20^. Xia *et al*. designed semi-supervised model called NetLapRLS which combines the information of the known drug-protein interaction network with genomic sequence data and chemical structure. In this model, the final result is predicted by the combination of the classifiers, and the method has achieved good performance because of utilizing the integrate information and unlabeled data^21^. He *et al*. proposed an effective model CCPMVFGC to calculate the degree of contextual correlation between pairwise vertex features. This model can learn a shared latent space from multi-view features, and use it to construct the interrelationship between pairs of vertices^22^. Hu *et al*. designed a novel GraphSE method to learning for patterns among drug side-effects (SEs), among drug sub-structures, and between multiple drug substructures and the SEs. This method can construct an attribute graph for each SE, which can effectively predict whether a drug will lead to a certain SE^23^. Chen *et al*. classified the current prediction model of drug-target interaction into network-based method and machine learning-based method and so on. In particular, they analyzed the supervised and semi-supervised methods in the adoption of negative samples in machine learning-based method^24^. Cao *et al*. proposed a new model for predicting DTIs which combines the protein information encoded by physicochemical and biochemical properties with drug molecules structures information encoded by MACCS substructure fingerings^25^. Chen *et al*. proposed the NRWRH model to identify DTIs based on the assumption that the framework of Random Walk and similar drugs target are often similar for the target protein^26^.

**Figure 1:**
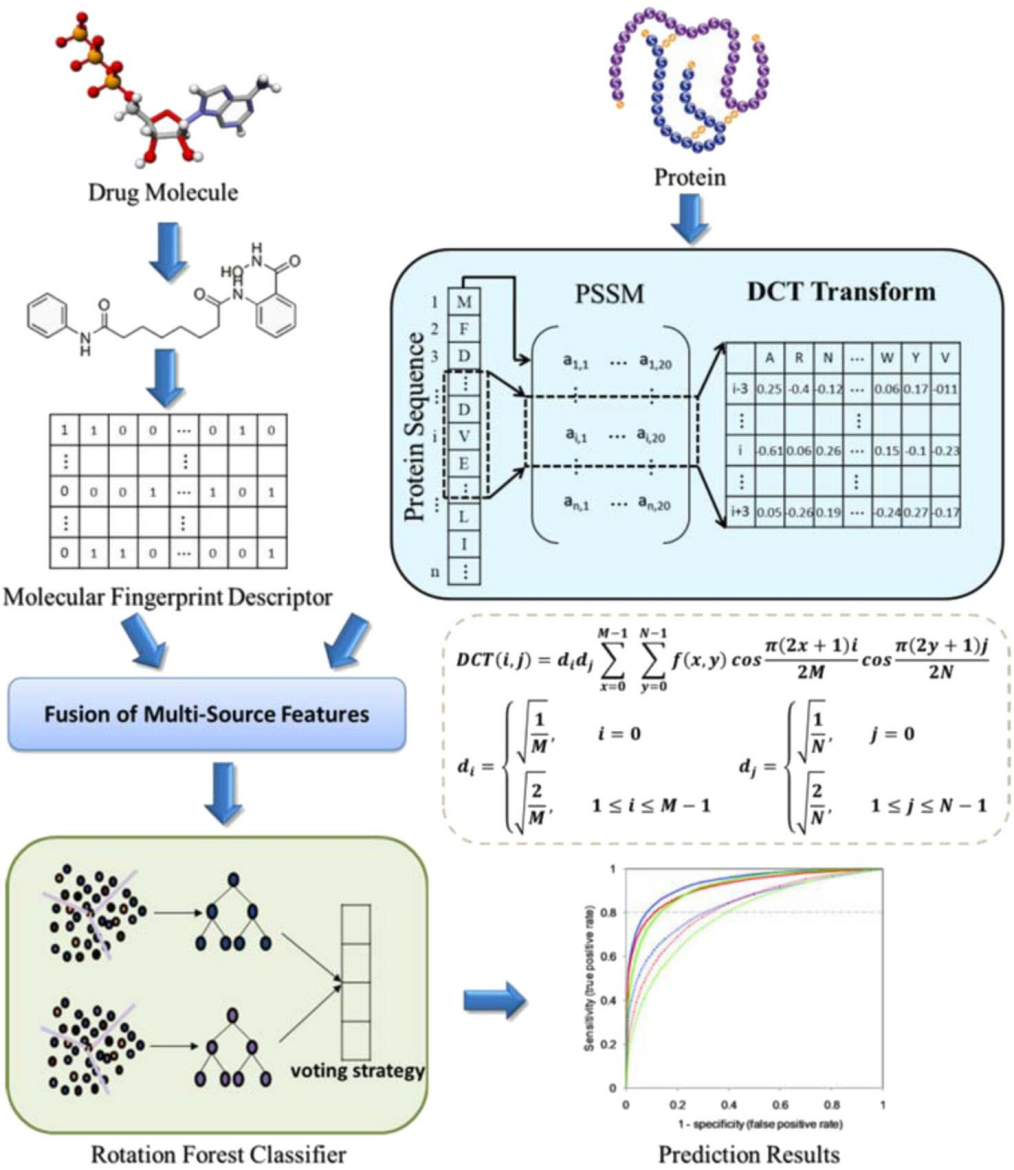
The workflow of the proposed model to predict potential drug-target interactions.

Under the premise of the theory that the interaction among drug and target protein depends largely on the chemical sub-structures of drug compound and the structure of target protein sequence^11,27–29^, we design a new *in silico* model to predict DTIs. Compared with the proposed methods, we introduce a protein sequence transformation method which can carry the information of biological evolution. In this method, the frequency of amino acid occurrences at different positions in multiple sequence comparisons is counted, and the conservative regions related to sequence evolution are found according to their probability distribution. Thus, similar parts between different sequences are found to infer their structural and functional similarities. The descriptors extracted by this method can not only reflect the position information of amino acids in the sequence, but also reflect the effects of mutations in amino acid sites during sequence evolution. Specifically, we firstly transform the protein sequence into numerical matrix that carries the information of biological evolution. Secondly, using Discrete Cosine Transform algorithm to extract its feature and combined with the corresponding chemical sub-structures as the feature vector. Finally, the Rotation Forest classifier is used to accurately predict the potential DTIs. We evaluate our model on *Enzymes*, *Ion Channels*, *GPCRs* and *Nuclear Receptors* datasets by the 5-fold CV method. Moreover, we compared the proposed model with the different feature extraction and classifiers models on the benchmark datasets. In the case study, the top 10 drug-target pairs with the highest predictive score were confirmed by SuperTarget database. Outstanding results show that the proposed model can effectively predict the relationship between drugs and targets, and can provide accurate candidates for biological experiments. The workflow of the proposed model is shown in Fig. 1.

## Materials and methods

### Benchmark datasets

In this work, the data for all DTIs were collected from DrugBank, SuperTarget, BRENDA, and KEGG BRITE by Yamanishi *et al*.^19^. These data is divided into four datasets including *Enzymes, Ion Channels, GPCRs* and *Nuclear Receptors*. The *Enzymes* dataset contains 445 drugs, 664 target proteins, and experimentally verified 2926 pairs of DTIs; The *Ion Channels* dataset contains 210 drugs, 204 target proteins, and experimentally verified 1476 pairs of DTIs; The *GPCRs* dataset contains 223 drugs, 95 target proteins, and experimentally verified 635 pairs of DTIs; The *Nuclear Receptors* dataset contains 54 drugs, 26 target proteins, and experimentally verified 90 pairs of DTIs. We take these known drug-target interactions as benchmark data and implement our experiments on this basis. The statistical information of drug target interaction is shown in Table 1.

**Table 1:** Statistics for the drug-target interactions.

Statistics	Enzyme	Ion Channel	GPCR	Nuclear Receptor
No. of drugs	445	210	223	54
No. of target proteins	664	204	95	26
No. of drug-target interactions	2926	1476	635	90

If the drug molecules and target proteins are regarded as nodes and the relationship between them is regarded as edges, we can build a network representing DTIs. After connecting the nodes representing the interaction of known drug targets, it can be seen that this network is sparse. In experiments, all pairs with drug-target interactions are considered to be positive samples, otherwise they are considered as negative samples. Take the *GPCRs* dataset for example, there are only 635 known DTIs but have 223 × 95 = 21185 edges in the network. It can be seen that the number (e.g. 21185-635 = 20550) of negative pairs is noticeably more than that of positive ones, which is about 97% of the sample space. Therefore, we use the down sampling algorithm to extract samples from unrelated drug-target pairs to construct the negative sample set. The number of these pairs is the same as that of the positive samples. Theoretically, these negative samples may contain drug-target pairs that have not been verified by experiments. However, from a probabilistic and statistical perspective, in such a large ratio of differences, the number of actual interaction pairs used as negative samples can be ignored.

### Drug molecular characterization

Studies show that molecular fingerprint of chemical sub-structures information can effectively characterize drug molecular information^30–32^. Therefore, molecular fingerprints are used herein to encode drug compounds in this paper. Specifically, this method encodes each molecular substructure as fingerprint and maps it into a corresponding Boolean vector. For a specific molecule, if it contains a molecular substructure, assign a value to 1 in the corresponding bit of the vector, otherwise 0. Although this method divides the molecule into individual fragments, it still retains the entire structure of the drug molecule. The ingenuity of this design is that it does not need the reasonable 3D conformation of molecules, so it will not accumulate errors from the description of molecular structure. In experiment, we adopt the chemical structure of the fingerprints set derived from the PubChem System. This drug fingerprint stores 881 molecular substructures, so the drug molecular descriptor used in this paper is an 881-dimensional vector.

### Numerical characterization of protein sequences

Protein sequences are usually stored in the form of letters, in which the number of letters is 20, representing 20 amino acids. In order to facilitate the processing of machine learning algorithm, we use Position-Specific Scoring Matrix (PSSM) to transform it into a numerical matrix^33,34^. The advantage of this strategy is that it can extract the biological evolutionary information carried in the protein sequence, which is conducive to deep mining. Suppose $PSSM=\{{\rho }_{i,j}:i=1\cdots L\,and\,j=1\cdots 20\}$, which is a matrix of *L × 20*. The number of *L* represents the length of the protein sequence, and the number of *20* indicates the kind of amino acids. So the PSSM can be expressed as:

1 $$PSSM=[\begin{array}{llll}{\ell }_{1,1} & {\ell }_{1,2} & \cdots  & {\ell }_{1,20}\\ {\ell }_{2,1} & {\ell }_{2,2} & \cdots  & {\ell }_{2,20}\\ \vdots  & \vdots  & \vdots  & \vdots \\ {\ell }_{L,1} & {\ell }_{L,2} & \cdots  & {\ell }_{L,20}\end{array}]$$

here ${\ell }_{i,j}$ means that the probability of the *ith* residue being mutated into type *j* during the procession of evolutionary in the protein from multiple sequence alignments.

In this work, one of the most effective and frequently-used application Position-Specific Iterated BLAST (PSI-BLAST) was used to generate PSSM. To achieve broad and high homologous sequences, its parameters *e-value* is set to 0.001, iteration is set to 3. Since all items in the *SwissProt* database have been strictly audited by experts, we use it as the comparison database for generating PSSM matrix in this work.

### Feature extraction

Feature extraction is one of the important steps in model construction. Effective feature descriptors can not only extract important information, but also can improve the performance of predictive model in predicting DTIs^35^. In this work, the Discrete Cosine Transform (DCT) is introduced to extract the features of the information representing the protein sequence from the PSSM. Due to the advantages of minimizing reconstruction errors and packing most of the information to a minimum of coefficients, the DCT only loses very little information during processing. The formula as follow:

2 $$DCT(i,j)={d}_{i}{d}_{j}\mathop{\sum }\limits_{x=0}^{M-1}\,\mathop{\sum }\limits_{y=0}^{N-1}f(x,y)\cos \,\frac{\pi (2x+1)i}{2M}\,\cos \,\frac{\pi (2y+1)j}{2N}\,0\le i\le M-1,\,0\le j\le N-1$$

where

3 $${d}_{i}=\{\begin{array}{c}\sqrt{\frac{1}{M}},i=0\\ \sqrt{\frac{2}{M}},1\le i\le M-1\end{array}$$

4 $${d}_{j}=\{\begin{array}{c}\sqrt{\frac{1}{N}},j=0\\ \sqrt{\frac{2}{N}},1\le j\le N-1\end{array}$$

$f(x,y)\in {P}^{N\times M}$ represents the PSSM matrix of *N* × *20* dimensions. After optimization, we selected the first 400 coefficients as the final feature descriptor representing the protein sequence.

### Classification prediction

In this work, we introduce Rotation Forest (RF) as a classifier for predicting DTIs. RF is a successful classifier proposed by Rodriguez *et al*.^36^. The basic idea of RF is to simultaneously build accurate and robust differential ensemble classifiers^37–39^. When the algorithm executes, RF first randomly divides the sample set, and then uses the transformation method to transform the subset to increase the difference between the subsets. Finally, the transformed subset is used to select samples to train different base classifiers.

Assume *S* denotes the sample set, $X={({x}_{1},{x}_{2},\ldots ,{x}_{n})}^{T}$ is the data and $Y={({y}_{1},{y}_{2},\ldots ,{y}_{n})}^{T}$ is the labels. Thus, the sample can be represented by $\{{x}_{i},{y}_{i}\}$. The forest is formed by *L* decision trees ${T}_{1},{T}_{2},\ldots ,{T}_{L}$. The algorithm can be described as follows:

**Algorithm:**
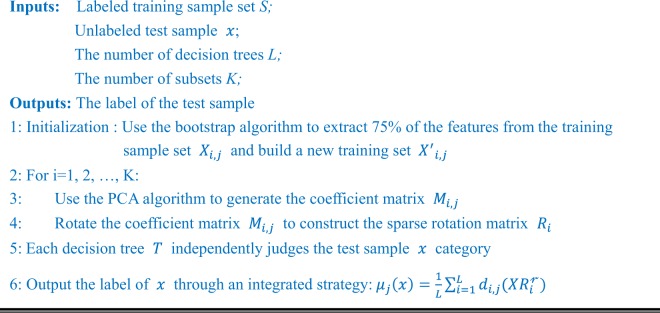
: The rotation forest classifier of the proposed model.

The sparse rotation matrix *R*_*i*_ can be expressed as follows:

5 $${R}_{i}=[\begin{array}{cccc}{r}_{i,1}^{(1)},\ldots ,{r}_{i,1}^{({C}_{1})} & 0 & \cdots  & 0\\ 0 & {r}_{i,2}^{(1)},\cdots ,{r}_{i,2}^{({C}_{2})} & \cdots  & 0\\ \vdots  & \vdots  & \ddots  & \vdots \\ 0 & 0 & \cdots  & {r}_{i,k}^{(1)},\ldots ,{r}_{i,k}^{({C}_{k})}\end{array}]$$

where $,{r}_{i,k}^{({C}_{k})}$ represents the coefficient in the matrix, ${R}_{i}^{r}$ represents the matrix obtained after reordering.

**Figure 2:**
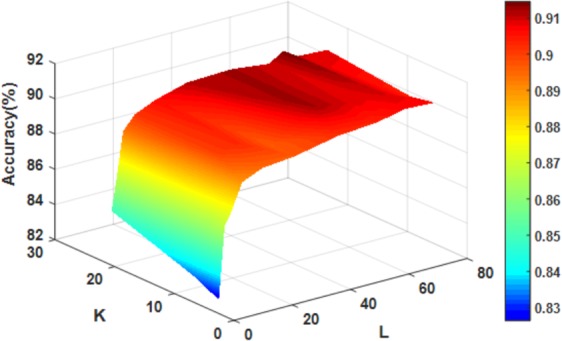
Accuracy surface obtained of rotation forest for optimizing parameter *K* and *L*.

In order to improve the performance of the model, we use the grid search method to optimize the parameters *K* and *L* of RF. Under different parameters, the accuracy of RF generation is shown in Fig. 2. As can be seen from the figure, with the increase of *K*, the value of accuracy gradually increased; with the increase of *L*, the value increases rapidly, then increases slowly, and finally decreases slightly. Considering the accuracy and time consumption, we finally chose the most suitable parameters of this experiment for k = 21 and L = 42.

## Results and Discussion

### Evaluation criteria

In this work, the evaluation criteria accuracy (Accu.), sensitivity (Sen.), precision (Prec.), and Matthews correlation coefficient (MCC) are utilized to estimate the performance of our model, and its formula is as follows:

6 $$Accu.=\frac{TP+TN}{TP+TN+FP+FN}$$

7 $$Sen.=\frac{TP}{TP+FN}$$

8 $$Prec.=\frac{TP}{TP+FP}$$

9 $$MCC=\frac{TP\times TN-FP\times FN}{\sqrt{(TP+FP)(TP+FN)(TN+FP)(TN+FN)}}$$

here TP, FP, TN and FN represent true positive, false positive, true negative and false negative, respectively. Furthermore, the Receiver Operating Characteristic (ROC)^40^ curve and the area under the curve (AUC) were also utilized to estimate the performance of the proposed model.

### Assessment of prediction ability

To be comparable, our classifier uses the same parameters when executed on four benchmark datasets. In experiment, the performance of our model is verified utilizing 5-fold cross-validation. This has the advantage of not only testing the model’s stability, but also avoiding over-fitting. Specifically, the whole dataset is split into five independent and equal-sized subsets, one of which serves as the test set and the remaining four as the training set. In the implementation, each take a different subset of the test set, loop 5 times.

**Figure 3:**
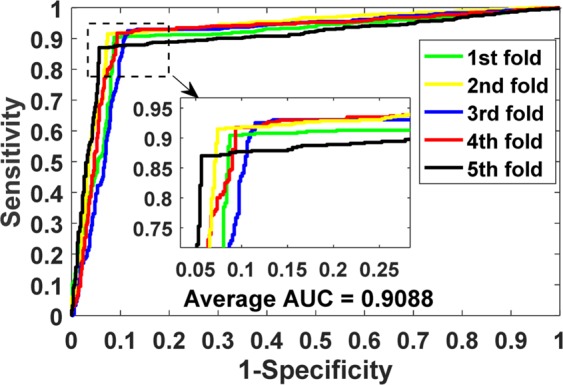
ROC curves performed by the proposed method on *Enzymes* dataset.

**Figure 4:**
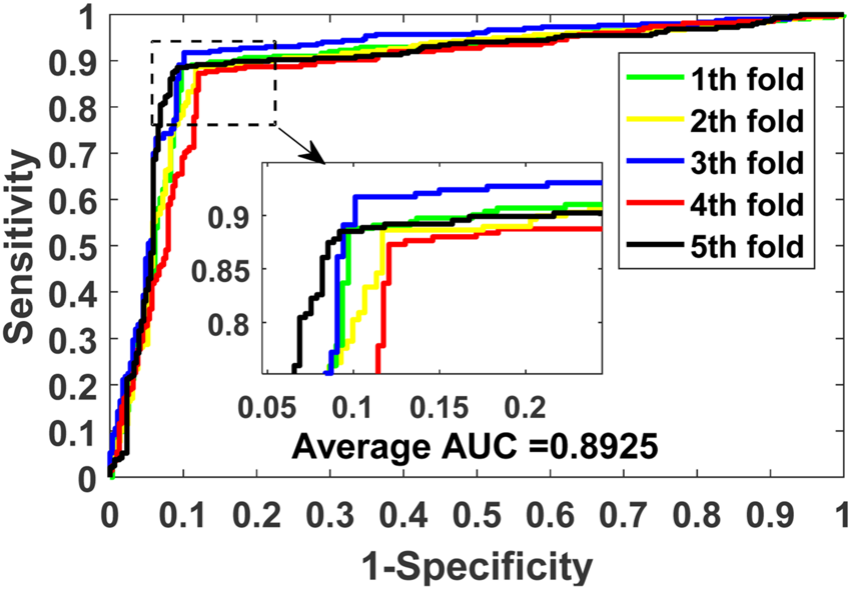
ROC curves performed by the proposed method on *Ion Channels* dataset.

**Figure 5:**
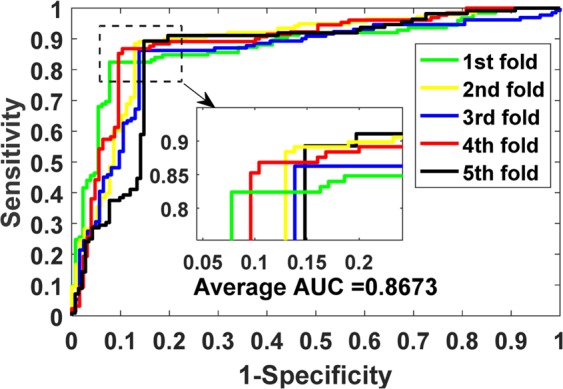
ROC curves performed by the proposed method on *GPCRs* dataset.

**Figure 6:**
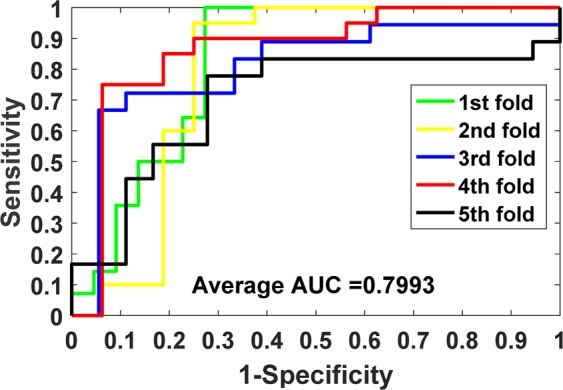
ROC curves performed by the proposed method on *Nuclear Receptors* dataset.

The prediction results of our model on benchmark datasets are summarized in Table 2. We obtained the average results of Accu., Prec., Sen., MCC and AUC of 0.9140, 0.9202, 0.9070, 0.8428 and 0.9088 when predicting drug-target interactions on *Enzymes* dataset. Their standard deviations are 0.0075, 0.0139, 0.0225, 0.0125 and 0.0116, respectively. We yielded the average evaluation criteria of 0.8919, 0.8928, 0.8899, 0.7836 and 0.8925 and their standard deviations are 0.0107, 0.0188, 0.0166, 0.0237 and 0.0140 when predicting drug-target interactions on *Ion Channels* dataset. In *GPCRs* dataset, we achieved the average results of Accu., Prec., Sen., MCC and AUC of 0.8724, 0.8799, 0.8632, 0.7454, 0.8673 and the standard deviations of 0.0066, 0.0337, 0.0272, 0.0134, 0.0181, respectively. We obtained the average evaluation criteria of 0.8111, 0.8040, 0.8346, 0.6328, 0.7993 and the standard deviations of 0.0412, 0.0944, 0.1160, 0.0817 and 0.0593 when predicting drug-target interactions on *Nuclear Receptors* dataset. The detailed results of 5-fold cross-validation on these four benchmark datasets can be seen in tables S1–S4 of the supplementary materials. Figures 3–6 show the ROC curves obtained by our model on those four benchmark datasets.

**Table 2:** Average 5-fold CV results obtained by our model on four benchmark datasets.

Dataset	Evaluation Criteria	Accu.	Prec.	Sen.	MCC	AUC
*Enzymes*	Average	0.9140	0.9202	0.9070	0.8428	0.9088
	Standard Deviation	0.0075	0.0139	0.0225	0.0125	0.0116
*Ion Channels*	Average	0.8919	0.8928	0.8899	0.7836	0.8925
	Standard Deviation	0.0107	0.0188	0.0166	0.0237	0.0140
*GPCRs*	Average	0.8724	0.8799	0.8632	0.7454	0.8673
	Standard Deviation	0.0066	0.0337	0.0272	0.0134	0.0181
*Nuclear Receptors*	Average	0.8111	0.8040	0.8346	0.6328	0.7993
	Standard Deviation	0.0412	0.0944	0.1160	0.0817	0.0593

### Comparison with Pseudo-AAC model and support vector machine model

In order to verify more clearly whether the DCT feature extraction algorithm and RF classifier can improve the performance of the model, we compare the proposed model with Pseudo-AAC model and SVM model. Specifically, we keep the other parts of the model unchanged, and only use Pseudo-AAC algorithm or SVM classifier to replace the feature extraction algorithm and classifier in the proposed model, and experiment in the same dataset.

The Pseudo-AAC algorithm can effectively extract the hydrophobic information in the protein sequence, but it does not retain the biological evolution information. Given a protein sequence *S*, the general form of Pseudo-AAC proposed by Chou *et al*.^41^ is defined as:

10 $$S={[{\varPhi }_{1},{\varPhi }_{2},\cdots ,{\varPhi }_{\mu },\cdots ,{\varPhi }_{L},]}^{T}$$

11 $${\varphi }_{\mu }=\,\{\begin{array}{c}{F}_{\mu }/({\sum }_{i=1}^{20}{F}_{i}+w{\sum }_{j=1}^{q}{\lambda }_{j}),\,(1\le \mu \le 20)\\ w{\theta }_{\mu -20}/({\sum }_{i=1}^{20}{F}_{i}+w{\sum }_{j=1}^{q}{\lambda }_{j}),\,(20+1\le \mu \le 20+q=L)\end{array}$$

here *L* is the length of the protein sequence, *F*_*i*_ is the normalized frequency of the amino acid in the protein, *w* is the weighting factor, and *λ*_*j*_ is the j-tier sequence correlation factor.

Table 3 summarizes the 5-fold CV results obtained by the Pseudo-AAC model on four benchmark datasets. It can be seen from the table that the Pseudo-AAC model has achieved the accuracy of 0.8450, 0.8296, 0.7425, 0.7000, precision of 0.8536, 0.8267, 0.7463, 0.6836, sensitivity of 0.8335, 0.8354, 0.7342, 0.7396, MCC of 0.6905, 0.6596, 0.4846, 0.3982, and AUC of 0.8435, 0.8314, 0.7531, 0.7259. Table 4 lists the 5-fold CV results obtained by the SVM model on four benchmark datasets. From the table we can see that the SVM model has achieved the accuracy of 0.8518, 0.8492, 0.7803, 0.6778, precision of 0.8479, 0.8499, 0.7753, 0.6726, sensitivity of 0.8578, 0.8492, 0.7944, 0.6905, MCC of 0.7040, 0.6984, 0.5640, 0.3605, and AUC of 0.8512, 0.8489, 0.7800, 0.6665.

**Table 3:** Average 5-fold CV results obtained by Pseudo-AAC model on four benchmark datasets.

Dataset	Evaluation Criteria	Accu.	Prec.	Sen.	MCC	AUC
*Enzymes*	Average	0.8450	0.8536	0.8335	0.6905	0.8435
	Standard Deviation	0.0085	0.0203	0.0120	0.0174	0.0140
*Ion Channels*	Average	0.8296	0.8267	0.8354	0.6596	0.8314
	Standard Deviation	0.0141	0.0182	0.0269	0.0286	0.0135
*GPCRs*	Average	0.7425	0.7463	0.7342	0.4846	0.7531
	Standard Deviation	0.0299	0.0321	0.0350	0.0588	0.0215
*Nuclear Receptors*	Average	0.7000	0.6836	0.7396	0.3982	0.7259
	Standard Deviation	0.0362	0.0714	0.0702	0.0826	0.0564

**Table 4:** Average 5-fold CV results obtained by SVM model on four benchmark datasets.

Dataset	Evaluation Criteria	Accu.	Prec.	Sen.	MCC	AUC
*Enzymes*	Average	0.8518	0.8479	0.8578	0.7040	0.8512
	Standard Deviation	0.0085	0.0184	0.0147	0.0168	0.0104
*Ion Channels*	Average	0.8492	0.8499	0.8492	0.6984	0.8489
	Standard Deviation	0.0139	0.0230	0.0108	0.0279	0.0154
*GPCRs*	Average	0.7803	0.7753	0.7944	0.5640	0.7800
	Standard Deviation	0.0218	0.0495	0.0425	0.0432	0.0339
*Nuclear Receptors*	Average	0.6778	0.6726	0.6905	0.3605	0.6665
	Standard Deviation	0.0421	0.0787	0.1151	0.0786	0.0718

**Figure 7:**
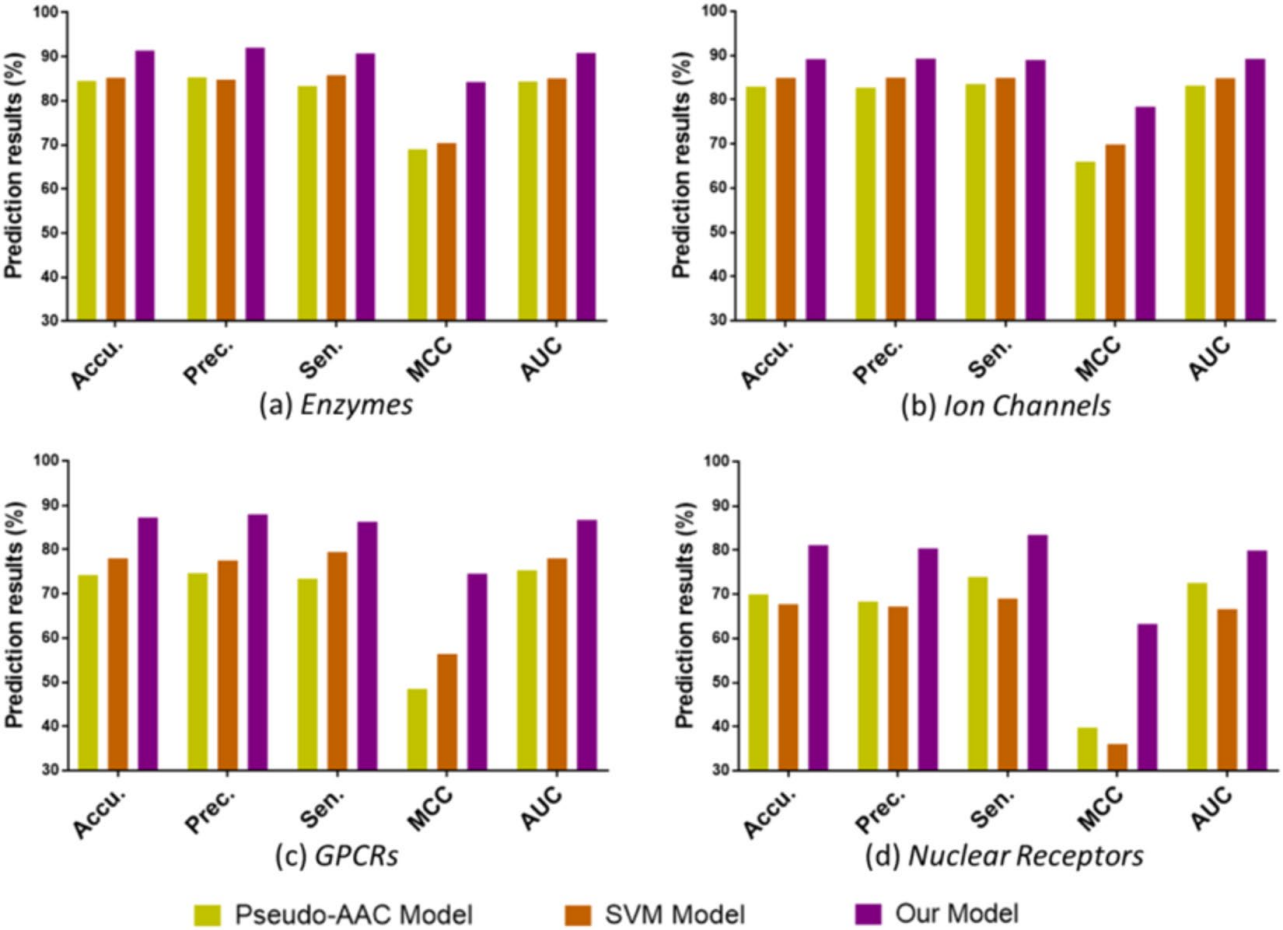
Comparison of experimental results of three models on the benchmark datasets. (**a**) The results of three models on *Enzymes* dataset using 5-flod CV. (**b**) The results of three models on *Ion Channels* dataset using 5-flod CV. (**c**) The results of three models on *GPCRs* dataset using 5-flod CV. (**d**) The results of three models on *Nuclear Receptors* dataset using 5-flod CV.

In order to facilitate the comparison, we present the results generated by the three models on the benchmark datasets in the form of histogram. From Fig. 7 we can see that the proposed model achieved the optimal results in all four datasets. In terms of accuracy, the proposed model is 0.0690 and 0.0622 higher than Pseudo-AAC model and SVM model respectively on *Enzymes* dataset, 0.0623 and 0.0427 on *Ion Channels* data set, 0.1299 and 0.0921 on *GPCRs* data set, 0.1111 and 0.1333 on *Nuclear Receptors* data set. The results show that the proposed model can predict the potential drug-target relationship more accurately than other models. In terms of AUC, the proposed model is 0.0653 and 0.0576 higher than Pseudo-AAC model and SVM model respectively on *Enzymes* dataset, 0.0611 and 0.0436 on *Ion Channels* data set, 0.1142 and 0.0873 on *GPCRs* data set, 0.0734 and 0.1328 on *Nuclear Receptors* data set. The results show that the proposed model has better overall performance than other models.

The excellent performance of the proposed model is mainly attributed to the following three points: (a) the model uses protein sequence characterization with biological evolution information and drug molecular characterization with molecular fingerprint information. This strategy can enrich the expression of drug target data information; (b) the DCT algorithm used in the model can effectively extract the hidden features in the drug-target data, and only lose a little information in the process of processing; c) the RF classifier used in the model can accurately and quickly classify drug-target data, thereby greatly improving the performance of the model.

### Comparison with state-of-the-art models

So far, there have been many state-of-the-art models to predict drug-target interactions and achieved good results. To fully evaluate the performance of the proposed model, we compare it with these state-of-the-art models on the benchmark datasets. Table 5 lists the values of AUC achieved by different models. It can be observed that the results obtained by our model have a significant improvement on benchmark datasets except *Nuclear Receptors* dataset. In the *Enzymes, Ion Channels* and *GPCRs* datasets, our model achieved the highest score, improving 0.0458, 0.0935, and 0.0003, respectively, over the next highest model. In the *Nuclear Receptor* dataset, our model achieved the third highest score, but it was also only 0.0567 lower than the highest SIMCOMP model.

**Table 5:** Comparison of other excellent models and the proposed model on four benchmark datasets in terms of the AUC.

Dataset	Our model	MLCLE^42^	KBMF2K^43^	AM-PSSM^44^	SIMCOMP^34^
*Enzymes*	**0.9088**	0.842	0.832	0.843	0.863
*Ion Channels*	**0.8925**	0.795	0.799	0.722	0.776
*GPCRs*	**0.8673**	0.850	0.857	0.839	0.867
*Nuclear Receptors*	0.7993	0.790	0.824	0.767	**0.856**

To further compare the performance of the proposed models, we evaluated the comparison results of Table 5 using statistical test. We make a hypothesis that there is no significant difference between our model and other models at 95% confidence level. If the *P-value* is lower than 0.05, we can believe that there are significant difference between the proposed model and other comparison models. As a result, we obtained the *P-value* of 0.044. These results show that the proposed model is significantly more competitive than other models and can effectively predict potential drug-target protein interactions.

### Case studies

In order to further evaluate the prediction ability of the proposed model for potential DTIs, we conducted the case studies. We train the model with all the positive samples in the benchmark datasets as the training set, and predict the score of the unknown associated drug-target pairs. For the top 10 drug-target pairs with the highest predicted scores, we put them into the SuperTarget database for verification. Table 6 summarizes the details of the top 10 drug-target pairs with the highest predicted scores. It can be seen from the table that 8 new drug-target pairs have been confirmed by the SuperTarget database. The results of case studies show that the proposed model can effectively predict the unknown association of drug-target pairs, and provide reliable candidates for biological experiments. It is worth noting that although the remaining two drug-target pairs have not been confirmed at present, the possibility of an association between them cannot be denied.

**Table 6:** Details of the top 10 drug-target pairs with the highest predicted scores.

Drug ID	Drug Name	Taregt Protein ID	Target Protein Name	Validation Source
D00049	Nikotinsaeure	hsa 8843	G109B_HUMAN	SuperTarget
D00348	Isotretinoino	hsa6256	RXRA_HUMAN	SuperTarget
D00437	Nifedipine Monohydrochloride	hsa1559	CP2C9_HUMAN	SuperTarget
D00139	Xanthotoxine	hsa1543	CP1A1_HUMAN	SuperTarget
D00585	Mifepristone	hsa2099	ESR1_HUMAN	SuperTarget
D00951	Medroxyprogesteroneacetate	hsa2099	ESR1_HUMAN	SuperTarget
D02340	Loxapinsuccinate	hsa1812	DRD1_HUMAN	SuperTarget
D00900	Monomethylhydrazine	hsa1020	CDK5_HUMAN	N/A
D03365	Transdermal Nicotine	hsa1137	ACHA4_HUMAN	SuperTarget
D00448	Methylphosphonothiolate	hsa10720	UDB11_HUMAN	N/A

## Conclusion

In this work, based on the assumption that the relationship between drugs and targets is largely influenced by the drug molecular structure and protein amino acid sequence, we proposed a novel model to predict DTIs by fusing protein sequence information and molecular fingerprint information. To improve the performance of the proposed model, we introduce the biological evolution information in the process of extracting protein features, and consider the excellent classifier in the process of feature classification. In the experiment, the proposed model was validated on four benchmark datasets including *Enzymes, Ion Channels, GPCRs* and *Nuclear Receptors*. Furthermore, we also compared with the different feature extraction model, classifier model and other state-of-the-art models. In the case study, 8 of the top 10 drug-target pairs predicted by our model were confirmed by relevant databases. These excellent results show that the proposed model is very suitable for predicting DTIs and can be an effective tool for providing reliable candidates for biological experiments. In the next research, we will focus on the feature extraction algorithm to further improve the performance of the model.

## Supplementary information

Supplementary Information.

## References

[1] Overington JP, Al-Lazikani B, Hopkins AL (2006). Opinion - How many drug targets are there?. Nature Reviews Drug Discovery.

[2] Rigden DJ, Fernández-Suárez XM, Galperin MY (2015). The 2016 database issue of Nucleic Acids Research and an updated molecular biology database collection. Nucleic acids research.

[3] Ezzat A, Zhao P, Wu M, Li XL, Kwoh CK (2017). Drug-Target Interaction Prediction with Graph Regularized Matrix Factorization. *IEEE/ACM Transactions on Computational Biology &*. Bioinformatics.

[4] Wang L, You Z-H, Huang D-S, Zhou F (2018). Combining High Speed ELM Learning with a Deep Convolutional Neural Network Feature Encoding for Predicting Protein-RNA Interactions. IEEE/ACM transactions on computational biology and bioinformatics.

[5] Gao, Z. G. *et al*. Ens-PPI: A Novel Ensemble Classifier for Predicting the Interactions of Proteins Using Autocovariance Transformation from PSSM. *Biomed Research International*, 8, 10.1155/2016/4563524 (2016). 10.1155/2016/4563524 PMC4942601 27437399

[6] Wang L (2017). An ensemble approach for large-scale identification of protein-protein interactions using the alignments of multiple sequences. Oncotarget.

[7] Yasuo, N., Nakashima, Y. & Sekijima, M. In *IEEE International Conference on Bioinformatics and Biomedicine (BIBM)*. 2018.

[8] Xia, L.-Y., Yang, Z.-Y., Zhang, H. & Liang, Y. Improved Prediction of Drug-Target Interactions Using Self-Paced Learning with Collaborative Matrix Factorization. *Journal of Chemical Information and Modeling* **59** (2019). 10.1021/acs.jcim.9b00408 31260620

[9] Coelho ED, Arrais JP, Oliveira JL (2016). Computational discovery of putative leads for drug repositioning through drug-target interaction prediction. PLoS computational biology.

[10] Peska L, Buza K, Koller J (2017). Drug-Target Interaction Prediction: a Bayesian Ranking Approach. Comput Methods Programs Biomed.

[11] Wang, L. *et al*. In *International Symposium on Bioinformatics Research and Applications*. 46–58 (Springer).

[12] Mousavian Z, Khakabimamaghani S, Kavousi K, Masoudi-Nejad A (2016). Drug–target interaction prediction from PSSM based evolutionary information. Journal of pharmacological and toxicological methods.

[13] Shaikh, N., Sharma, M. & Garg, P. An improved approach for predicting drug-target interaction: Proteochemometrics to molecular docking. *Molecular Biosystems* **12** (2016). 10.1039/c5mb00650c 26822863

[14] Rayhan F (2017). iDTI-ESBoost: identification of drug target interaction using evolutionary and structural features with boosting. Scientific reports.

[15] Vilar S (2016). Computational drug target screening through protein interaction profiles. Scientific reports.

[16] Wang L (2018). RFDT: A Rotation Forest-based Predictor for Predicting Drug-Target Interactions Using Drug Structure and Protein Sequence Information. Current Protein & Peptide Science.

[17] Peón A, Naulaerts S, Ballester PJ (2017). Predicting the reliability of drug-target interaction predictions with maximum coverage of target space. Scientific reports.

[18] Chen, H. & Zhang, Z. A Semi-Supervised Method for Drug-Target Interaction Prediction with Consistency in Networks. *Plos One* **8**, 10.1371/journal.pone.0062975 (2013). 10.1371/journal.pone.0062975 PMC3646965 23667553

[19] Yamanishi Y, Araki M, Gutteridge A, Honda W, Kanehisa M (2008). Prediction of drug-target interaction networks from the integration of chemical and genomic spaces. Bioinformatics.

[20] He, T., Chan, K. C. & Yang, L. In *IEEE/WIC/ACM International Conference on Web Intelligence (WI)*. 401–406 (IEEE). 2018

[21] Xia, Z., Wu, L.-Y., Zhou, X. & Wong, S. T. C. Semi-supervised drug-protein interaction prediction from heterogeneous biological spaces. *Bmc Systems Biology* **4**, 10.1186/1752-0509-4-s2-s6 (2010). 10.1186/1752-0509-4-S2-S6 PMC2982693 20840733

[22] He, T., Liu, Y., Ko, T. H., Chan, K. C. & Ong, Y.-S. Contextual Correlation Preserving Multiview Featured Graph Clustering. *IEEE transactions on cybernetics* **1–1** (2019). 10.1109/TCYB.2019.2926431 31329151

[23] Hu, P. *et al*. In *IEEE International Conference on Bioinformatics and Biomedicine (BIBM)*. 1163–1169 (IEEE). 2018

[24] Chen X (2016). Drug–target interaction prediction: databases, web servers and computational models. Briefings in bioinformatics.

[25] Cao D-S (2012). Large-scale prediction of drug-target interactions using protein sequences and drug topological structures. Analytica Chimica Acta.

[26] Chen X, Yan G-Y (2010). NRWRH for Drug Target Prediction. Computational Systems Biology.

[27] Zhang W, Chen Y, Li D (2017). Drug-Target Interaction Prediction through Label Propagation with Linear Neighborhood Information. Molecules.

[28] Zong, N., Kim, H., Ngo, V. & Harismendy, O. Deep Mining Heterogeneous Networks of Biomedical Linked Data to Predict Novel Drug-Target Associations. *Bioinformatics* **33** (2017). 10.1093/bioinformatics/btx160 PMC5860112 28430977

[29] Wang L (2019). LMTRDA: Using logistic model tree to predict MiRNA-disease associations by fusing multi-source information of sequences and similarities. PLoS computational biology.

[30] Wu Z (2017). SDTNBI: an integrated network and chemoinformatics tool for systematic prediction of drug–target interactions and drug repositioning. Briefings in Bioinformatics.

[31] Peng L, Liao B, Zhu W, Li Z, Li K (2017). Predicting Drug-Target Interactions With Multi-Information Fusion. IEEE Journal of Biomedical & Health Informatics.

[32] Ezzat A, Wu M, Li XL, Kwoh CK (2017). Drug-Target Interaction Prediction using Ensemble Learning and Dimensionality Reduction. Methods.

[33] Wang L, Wang H-F, Liu S-R, Yan X, Song K-J (2019). Predicting Protein-Protein Interactions from Matrix-Based Protein Sequence Using Convolution Neural Network and Feature-Selective Rotation Forest. Scientific reports.

[34] Öztürk H, Ozkirimli E, Özgür A (2016). A comparative study of SMILES-based compound similarity functions for drug-target interaction prediction. BMC Bioinformatics.

[35] Wang L (2018). Using Two-dimensional Principal Component Analysis and Rotation Forest for Prediction of Protein-Protein Interactions. Scientific reports.

[36] Rodriguez JJ, Kuncheva LI (2006). Rotation forest: A new classifier ensemble method. Ieee Transactions on Pattern Analysis and Machine Intelligence.

[37] Wang L (2017). Advancing the prediction accuracy of protein-protein interactions by utilizing evolutionary information from position-specific scoring matrix and ensemble classifier. Journal Of Theoretical Biology.

[38] Xia J, Du P, He X, Chanussot J (2013). Hyperspectral remote sensing image classification based on rotation forest. IEEE Geoscience and Remote Sensing Letters.

[39] Lu H, Meng Y, Yan K, Gao Z (2019). Kernel principal component analysis combining rotation forest method for linearly inseparable data. Cognitive Systems Research.

[40] Hajian-Tilaki K (2013). Receiver operating characteristic (ROC) curve analysis for medical diagnostic test evaluation. Caspian journal of internal medicine.

[41] Chou KC (2011). Some remarks on protein attribute prediction and pseudo amino acid composition. Journal of Theoretical Biology.

[42] Pliakos, K., Vens, C. & Tsoumakas, G. Predicting drug-target interactions with multi-label classification and label partitioning. *IEEE/ACM transactions on computational biology and bioinformatics* (2019). 10.1109/TCBB.2019.2951378 31689203

[43] Gonen M (2012). Predicting drug-target interactions from chemical and genomic kernels using Bayesian matrix factorization. Bioinformatics.

[44] Mousavian Z, Khakabimamaghani S, Kavousi K, Masoudi-Nejad A (2015). Drug-Target Interaction Prediction from PSSM based Evolutionary Information. J Pharmacol Toxicol Methods.

