# Supplementary material for: Incorporating chemical sub-structures and protein evolutionary information for inferring drug-target interactions
Source: Sci Rep. 2020 Apr 20;10:6641. doi: 10.1038/s41598-020-62891-2 (PMC7171114; doi:10.1038/s41598-020-62891-2)
Supplement: Supplementary file 1 — Supplementary Information. [file 41598_2020_62891_MOESM1_ESM.docx]

**Table S1.** 5-fold CV results obtained by our model on *Enzymes* dataset

| **Test set** | **Accu.** | **Prec.** | **Sen.** | **MCC** | **AUC** |
| --- | --- | --- | --- | --- | --- |
| 1 | 0.9077 | 0.9105 | 0.9043 | 0.8324 | 0.9010 |
| 2 | 0.9197 | 0.9258 | 0.9133 | 0.8522 | 0.9275 |
| 3 | 0.9239 | 0.9219 | 0.9296 | 0.8593 | 0.9009 |
| 4 | 0.9120 | 0.9033 | 0.9175 | 0.8394 | 0.9129 |
| 5 | 0.9070 | 0.9392 | 0.8703 | 0.8308 | 0.9017 |
| **Average** | **0.9140±0.0075** | **0.9202±0.0139** | **0.9070±0.0225** | **0.8428±0.0125** | **0.9088±0.0116** |

**Table S2.** 5-fold CV results obtained by our model on *Ion Channels* dataset

| **Test set** | **Accu.** | **Prec.** | **Sen.** | **MCC** | **AUC** |
| --- | --- | --- | --- | --- | --- |
| 1 | 0.8949 | 0.9112 | 0.8878 | 0.7898 | 0.8922 |
| 2 | 0.8847 | 0.8863 | 0.8863 | 0.7694 | 0.8880 |
| 3 | 0.9068 | 0.9026 | 0.9175 | 0.8135 | 0.9142 |
| 4 | 0.8763 | 0.8633 | 0.8727 | 0.7516 | 0.8754 |
| 5 | 0.8970 | 0.9007 | 0.8850 | 0.7937 | 0.8928 |
| **Average** | **0.8919±0.0107** | **0.8928±0.0188** | **0.8899±0.0166** | **0.7836±0.0237** | **0.8925±0.0140** |

**Table S3.** 5-fold CV results obtained by our model on *GPCRs* dataset

| **Test set** | **Accu.** | **Prec.** | **Sen.** | **MCC** | **AUC** |
| --- | --- | --- | --- | --- | --- |
| 1 | 0.8740 | 0.9115 | 0.8240 | 0.7510 | 0.8752 |
| 2 | 0.8780 | 0.8905 | 0.8841 | 0.7543 | 0.8817 |
| 3 | 0.8622 | 0.8692 | 0.8626 | 0.7242 | 0.8465 |
| 4 | 0.8780 | 0.9016 | 0.8527 | 0.7572 | 0.8840 |
| 5 | 0.8701 | 0.8265 | 0.8929 | 0.7406 | 0.8491 |
| **Average** | **0.8724±0.0066** | **0.8799±0.0337** | **0.8632±0.0272** | **0.7454±0.0134** | **0.8673±0.0181** |

**Table S4.** 5-fold CV results obtained by our model on *Nuclear Receptors* dataset

| **Test set** | **Accu.** | **Prec.** | **Sen.** | **MCC** | **AUC** |
| --- | --- | --- | --- | --- | --- |
| 1 | 0.8056 | 0.6842 | 0.9286 | 0.6404 | 0.8279 |
| 2 | 0.8611 | 0.8261 | 0.9500 | 0.7242 | 0.7938 |
| 3 | 0.8056 | 0.9231 | 0.6667 | 0.6361 | 0.8086 |
| 4 | 0.8333 | 0.8500 | 0.8500 | 0.6625 | 0.8625 |
| 5 | 0.7500 | 0.7368 | 0.7778 | 0.5008 | 0.7037 |
| **Average** | **0.8111±0.0412** | **0.8040±0.0944** | **0.8346±0.1160** | **0.6328±0.0817** | **0.7993±0.0593** |
